# Supplementary material for: 2D Titanium carbide printed flexible ultrawideband monopole antenna for wireless communications
Source: Nat Commun. 2023 Jan 17;14:278. doi: 10.1038/s41467-022-35371-6 (PMC9845342; doi:10.1038/s41467-022-35371-6)
Supplement: Supplementary file 1 — Supplementary Information [file 41467_2022_35371_MOESM1_ESM.pdf]

2D Titanium Carbide Printed Flexible Ultrawideband Monopole Antenna for Wireless Communications

Weiwei Zhao,[1,†] Hao Ni,[2,†] Chengbo Ding,[1] Leilei Liu,[2,*] Qingfeng Fu,[2] Feifei Lin,[1] Feng Tian,[3] Pin Yang,[1] Shujuan Liu,[1] Wenjun He,[1] Xiaoming Wang,[1] Wei Huang,[1,4,*] Qiang Zhao[1,2,*]

[1] State Key Laboratory of Organic Electronics and Information Displays & Jiangsu Key Laboratory for Biosensors, Institute of Advanced Materials (IAM), Nanjing University of Posts & Telecommunications, 9 Wenyuan, Nanjing 210023, P. R. China

[2] College of Electronic and Optical Engineering & College of Flexible Electronics (Future Technology), National and Local Joint Engineering Laboratory of RF Integration and Micro-Assembly Technology, Nanjing University of Posts & Telecommunications, 9 Wenyuan, Nanjing 210023, P. R. China

[3] Key Lab of Broadband Wireless Communication and Sensor Network Technology, Nanjing University of Posts and Telecommunications, 9 Wenyuan, Nanjing 210023, P. R. China

[4] Frontiers Science Center for Flexible Electronics (FSCFE), MIIT Key Laboratory of Flexible Electronics (KLoFE), Northwestern Polytechnical University, Xi'an 710072, P. R. China

[†] These authors contributed equally: Weiwei Zhao and Hao Ni

E-mail addresses: liull@njupt.edu.cn (L. Liu), provost@nwpu.edu.cn (W. Huang), iamqzhao@njupt.edu.cn (Q. Zhao)

Table of Contents

Supplementary Figures…………………………………………………..…….S3

- Supplementary Figures 1−31

Supplementary Tables…………………………………………….…..…….S40

- Supplementary Tables 1−3

Supplementary References……………………………………….…..….….S44

Supplementary Figures

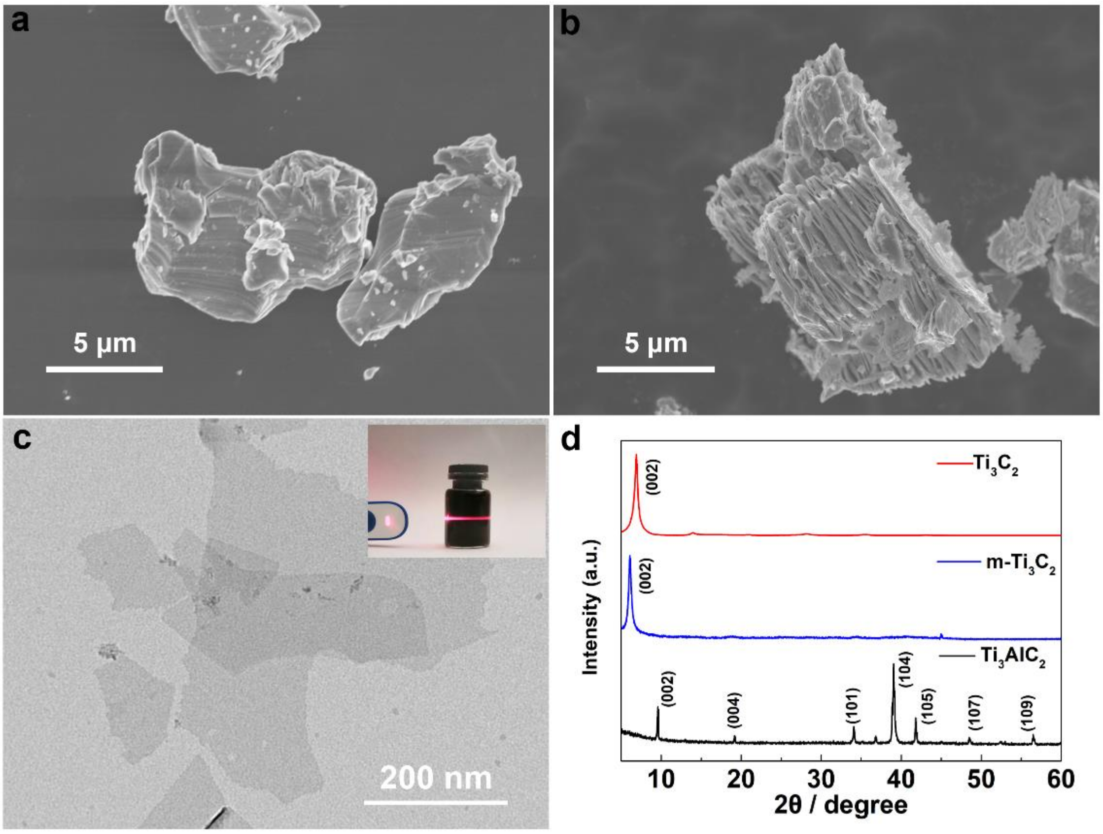

Supplementary Fig. 1 | The synthesis of Ti_3C_2 nanosheets. a SEM image of the commercial Ti_3AlC_2 bulks. **b** SEM image of m-Ti_3C_2 bulks. **c** TEM image of the ultrathin Ti_3C_2 nanosheets. Inset: Tyndall effect of Ti_3C_2 colloids. **d** XRD patterns of Ti_3AlC_2 bulks, m-Ti_3C_2 bulks, and Ti_3C_2 nanosheets.

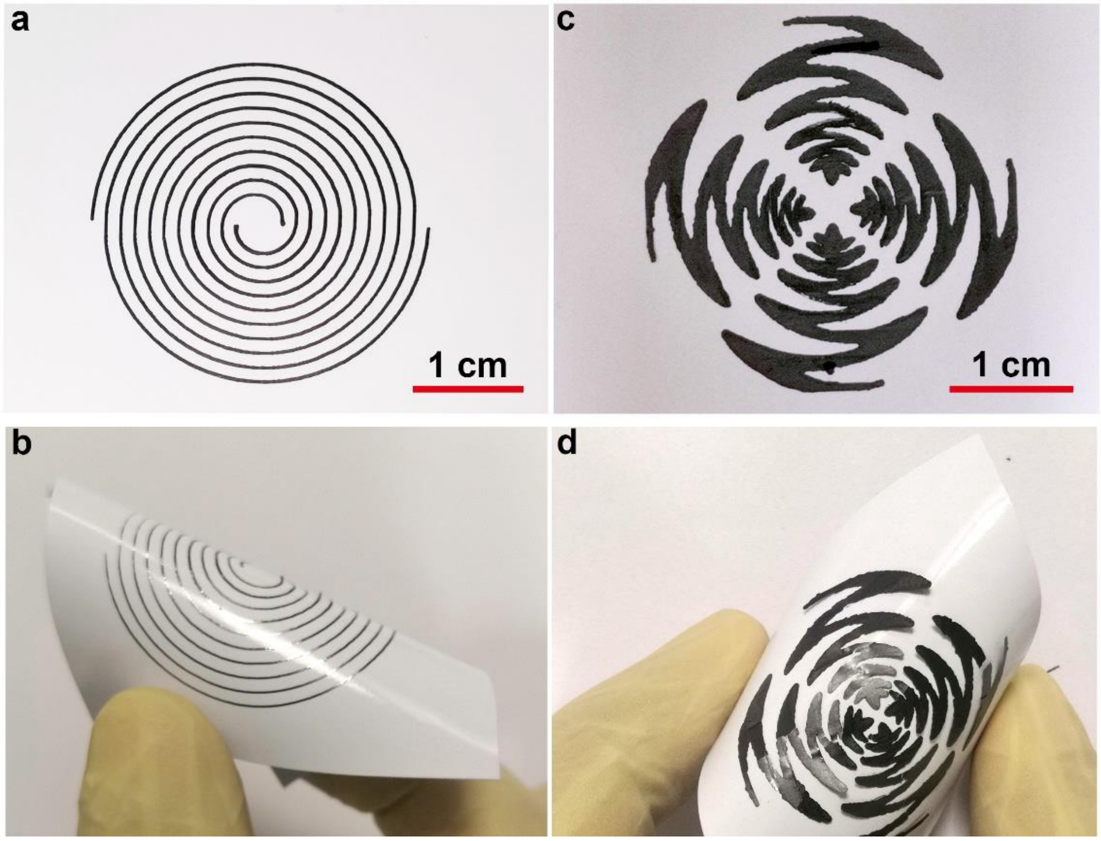

Supplementary Fig. 2 | The extrusion printing technology for flexible patterned patch antenna. a-b Archimedes spiral antenna. **c-d** Ultrawideband planar sinusoidal antenna.

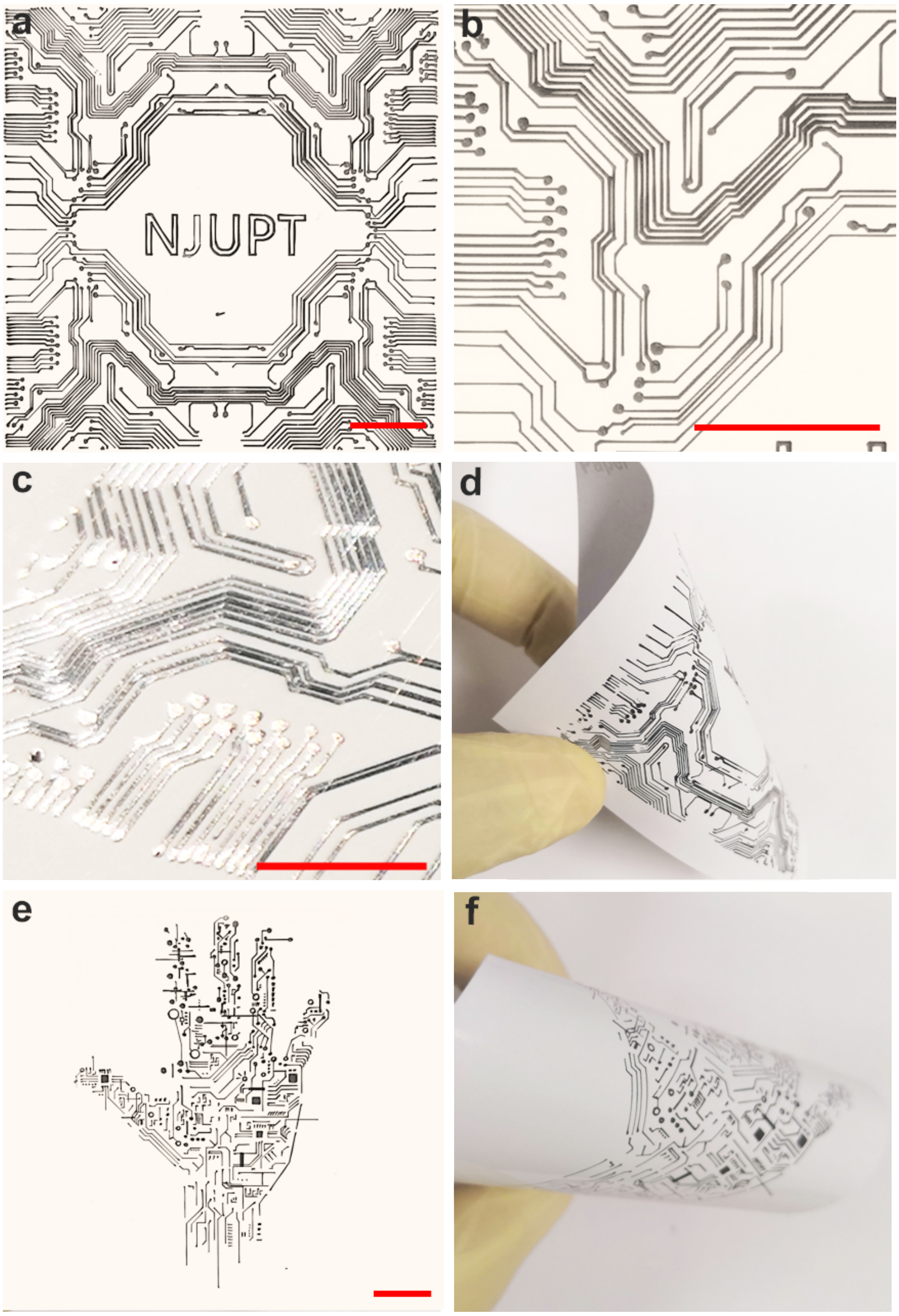

Supplementary Fig. 3 | The extrusion printing technology for patterned electronic circuits. a A complex pattern printed with Ti_3C_2 inks on the photographic paper. **b** Local enlarged drawings. **c** The tiny conductive path with metallic luster under the sun. **d** Photographs in flexible states. **e** The precise electronic circuits. **f** The electronic circuits in flexible states. The scale bar is 1 cm.

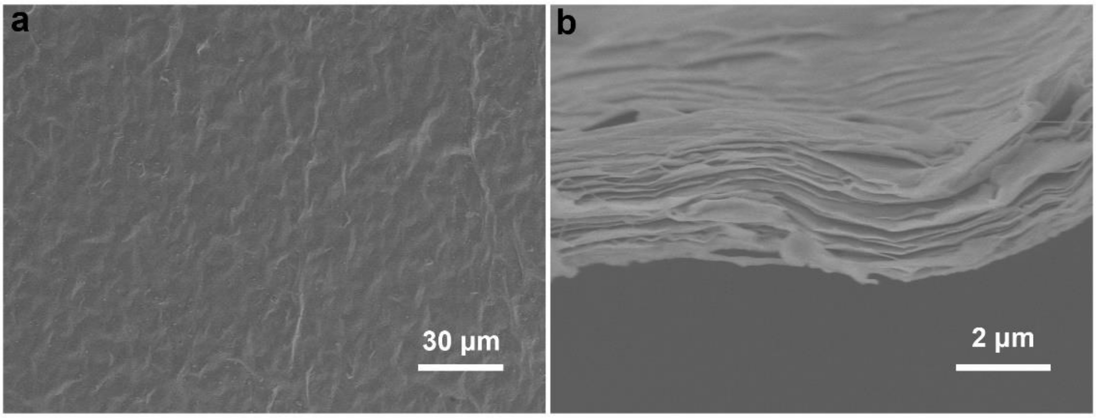

Supplementary Fig. 4 | SEM image of the multilayer Ti_3C_2 film. a Top-view SEM image. **b** Side-view SEM image.

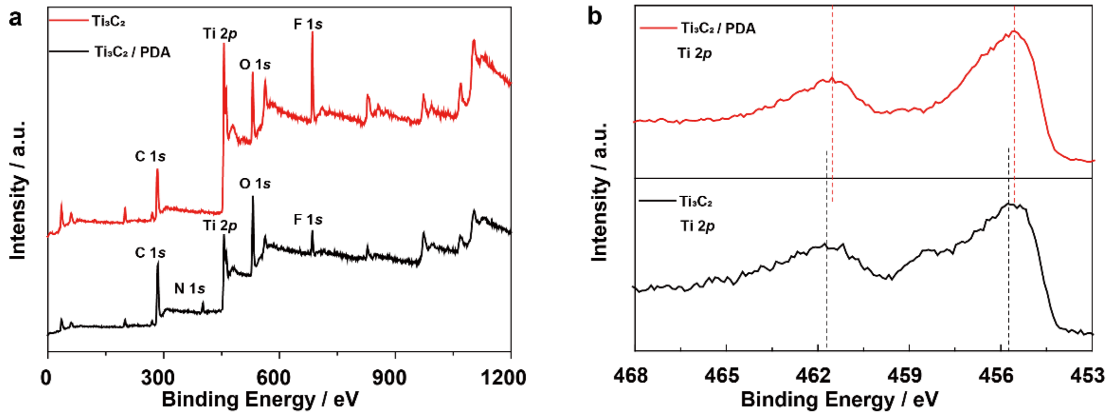

Supplementary Fig. 5 | XPS spectra of Ti_3C_2 and Ti_3C_2/PDA. a Full spectrum analysis. **b** Ti 2*p* spectra.

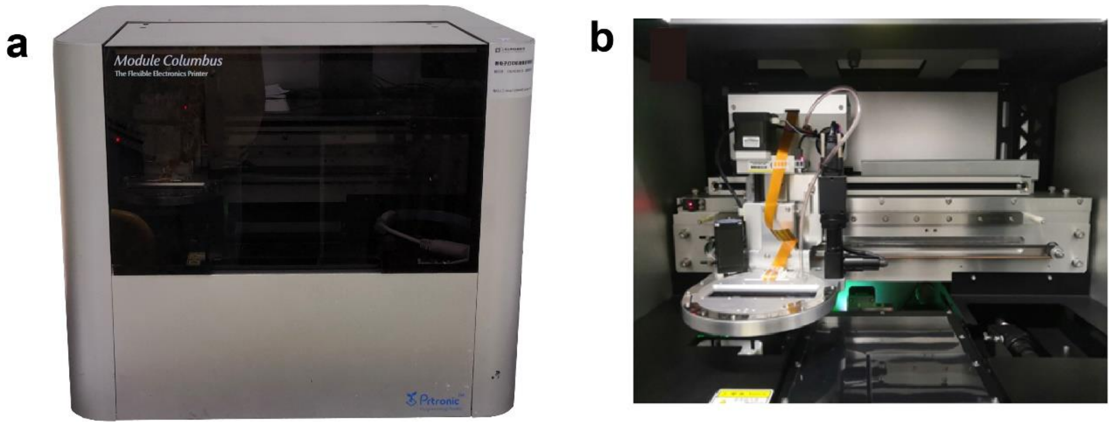

Supplementary Fig. 6 | Microelectronic printer for extrusion printing technology.

a The external photograph. **b** The internal structure.

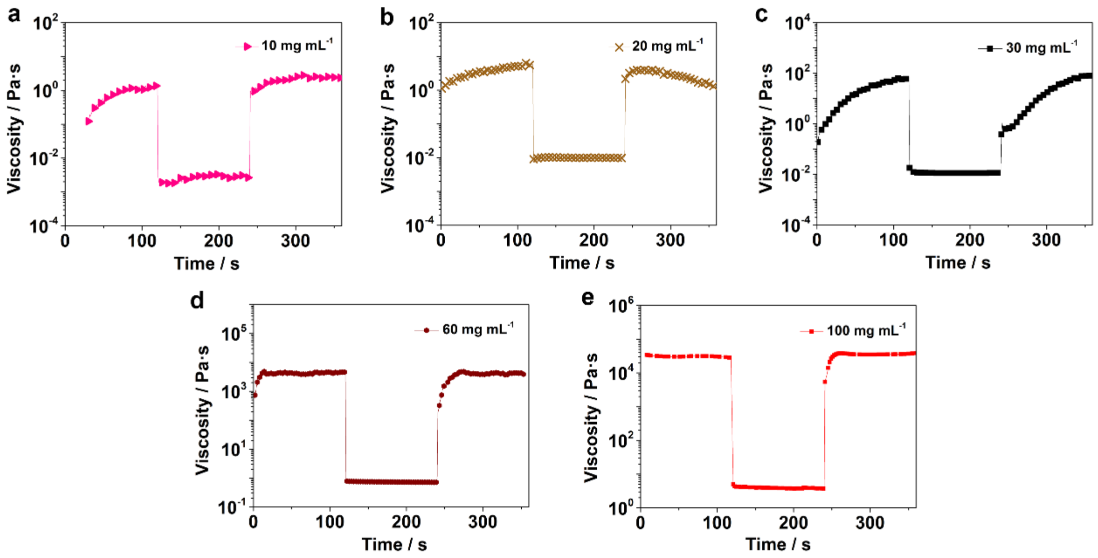

Supplementary Fig. 7 | Viscoelastic curves of Ti_3C_2 inks with different concentrations. **a**10 mg mL^{-1}, **b** 20 mg mL^{-1}, **c** 30 mg mL^{-1}, **d** 60 mg mL^{-1}, **e** 100 mg mL^{-1}.

The thixotropy curve shows that the viscosity of the ink with higher concentration can be recovered in a short time after the shear rate changes from high to low. It is used to simulate the state after the ink is extruded.

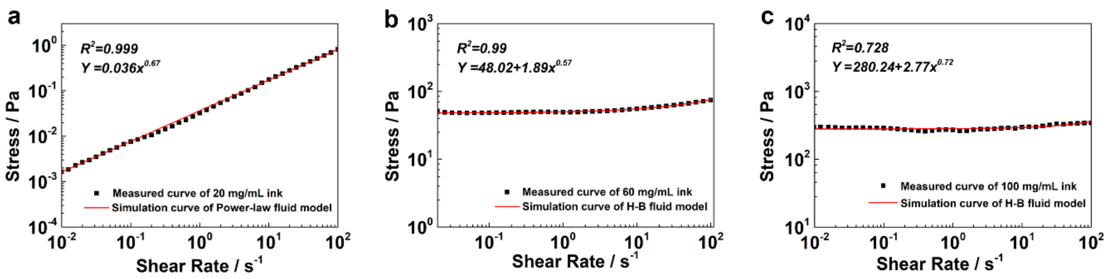

Supplementary Fig. 8 | Hershel-Bulkley fluid model fitting of Ti_3C_2 inks at the concentrations of 20 mg mL^{-1} (**a**), 60 mg mL^{-1} (**b**), 100 mg mL^{-1} (**c**). The ink of 60 mg mL^{-1} has the appropriate yield stress at 48 Pa.

For inks with the concentrations of 60 and 100 mg mL^{-1}, the Hershel-Bulkley fluid model is used to fit the yield stress.[1] It is simulated by the equation $\tau = \tau y(1+(\gamma/\gamma C)p)$, where τ is the measured shear stress, τy is the yield stress, γ is the applied shear rate, γC is the critical shear rate, and p is a dimensionless constant. For instance, the yield stress is 48 and 280 Pa for the inks of 60 and 100 mg mL^{-1}, respectively. In order to achieve good control of the Ti_3C_2 inks, we use a small printing aperture (60 μm diameter), and find that the yield stress of 48 Pa is the most suitable parameter in the actual printing process. Due to the small aperture and the fast-drying property of pure water-based ink, the stress of 280 Pa is easy to block the pores. As the concentration is lower than 30 mg mL^{-1}, the yield stress is less than 1 Pa, and thus the ink flow will lead to decreased accuracy.

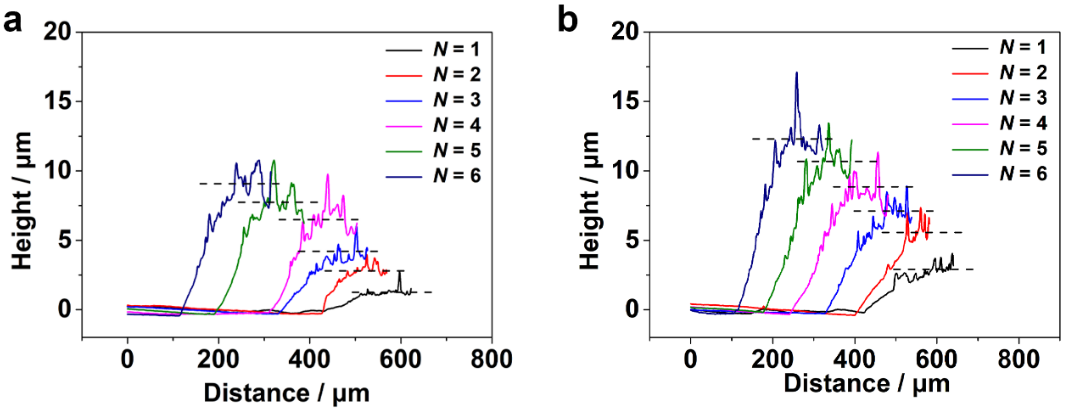

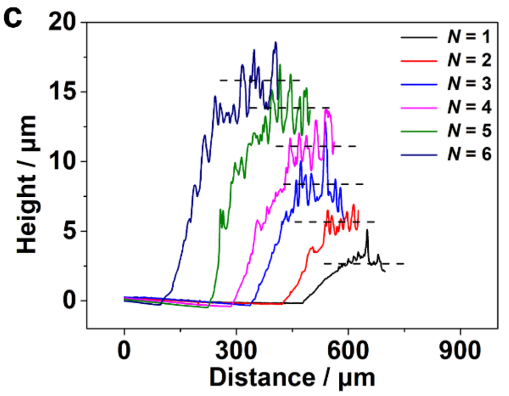

d

t (µm) \ *P* (kPa) / *L* (*N*)	50	60	70
1	1.2 ± 0.2	2.5 ± 0.6	2.8 ± 0.5
2	2.7 ± 0.3	4.0 ± 1.5	5.2 ± 1.3
3	3.8 ± 0.5	5.4 ± 1.7	7.3 ± 1.5
4	5.9 ± 1.3	8.3 ± 1.0	10.5 ± 1.6
5	7.7 ± 1.0	10.3 ± 1.2	12.7 ± 1.6
6	8.4 ± 1.4	12.1 ± 1.2	14.1 ± 1.9

Supplementary Fig. 9 | The thicknesses of Ti_3C_2 film measured by the step profiler. The corresponding height profiles of Ti_3C_2 films with different layers at a certain pressure of (**a**) 50 kPa, (**b**) 60 kPa, (**c**) 70 kPa. **d** Thickness of Ti_3C_2 films with different printing layers and pressures.

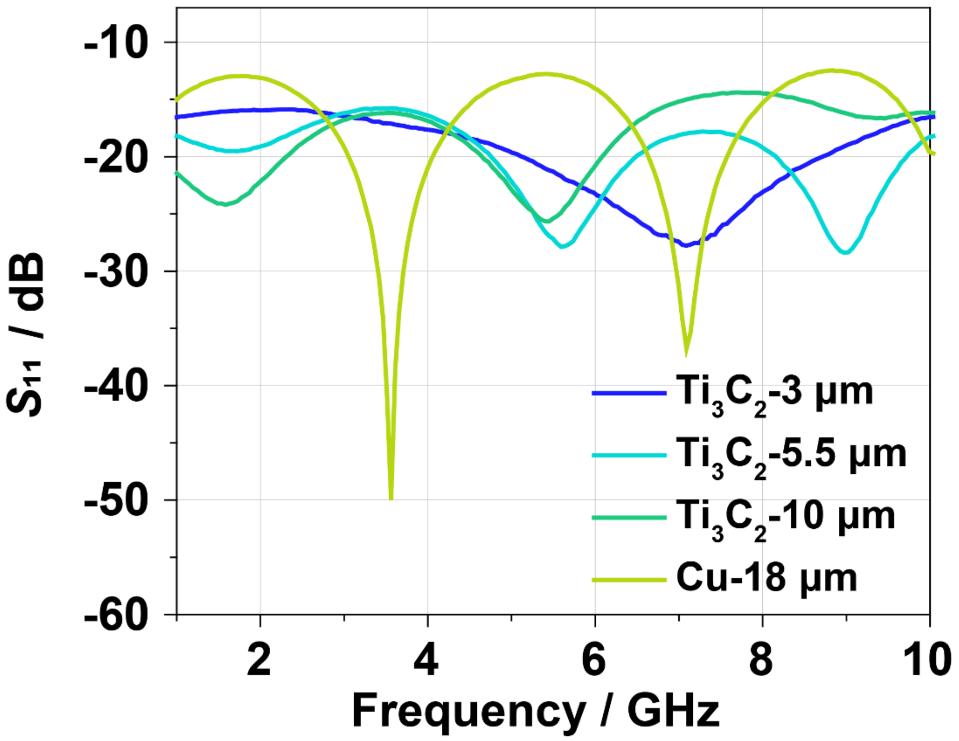

Supplementary Fig. 10 | Reflection coefficient S_{11} of the transmission lines with different thickness at the frequency from 1 to 10 GHz.

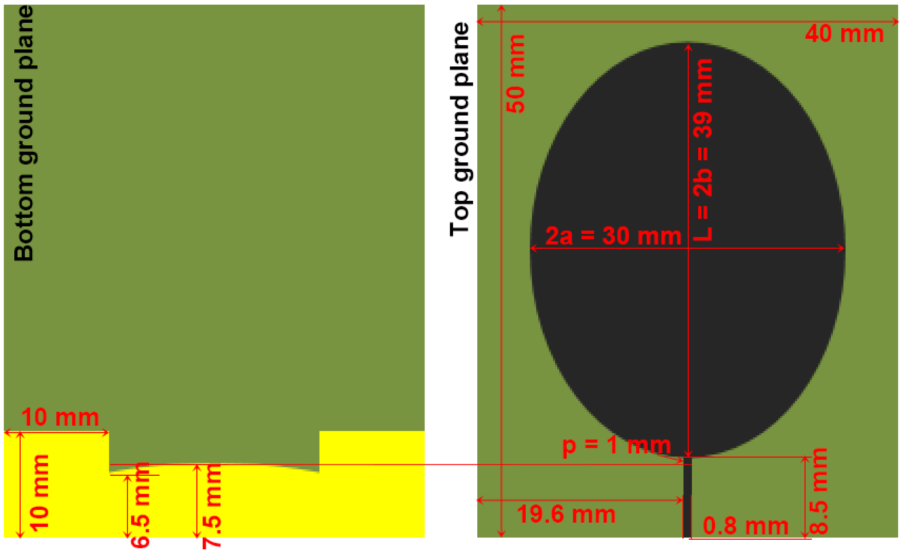

Supplementary Fig. 11 | The dimension and geometry of ultrawideband elliptical Ti_3C_2 monopole antennas working in the frequency of 1.7-4.0 GHz.

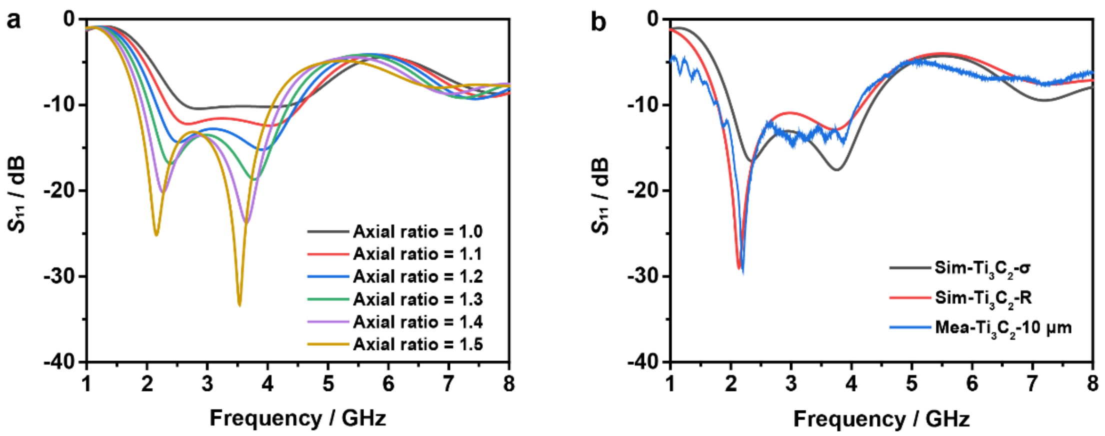

Supplementary Fig. 12 | The design principles of the ultrawideband elliptical Ti_3C_2 monopole antennas. a Simulated S_{11} of the Ti_3C_2 antennas with different axial ratios and measured S_{11} of the Ti_3C_2 antennas with axial ratio of 1.3. **b** Comparison of the measured and simulated S_{11} of the Ti_3C_2 antennas by the parameters of conductivity and sheet resistance.

In 1992, Honda first proposed an ultrawideband circular monopole antenna with an extraordinary impedance bandwidth and omnidirectional radiation performance due to the symmetrical current distribution on the disk.[2] In comparison with the circular patch, the elliptical patch has similar symmetrical currents and improved impedance bandwidth due to the better impedance matching performance.[3]

In order to obtain the widest impedance bandwidth, the axial ratio of the elliptical Ti_3C_2 antennas is first analyzed and the simulated S_{11} is shown in Supplementary Fig. 12a. When the axial ratio of the ellipse is less than 1.3, the relative bandwidth of the antenna widens with the increase of the axial ratio. S_{11} of the antenna is greater than -10 dB in 3-4 GHz. However, when the axial ratio of the ellipse is greater than 1.3, the

relative bandwidth of the antenna is almost unchanged. Considering the miniaturization requirement of the antenna, the axial ratio of the ellipse is set as 1.3.

The material system and full-wave simulation are combined based on sheet resistance for the first time to accurately predict the performance of Ti_3C_2 antennas (Supplementary Fig. 12b). In comparison with conductivity, the sheet resistance is more accurate for full-wave simulation in Ti_3C_2 microwave performance analysis. The actual bandwidth of Ti_3C_2-10 μm antennas is consistent with the simulated bandwidth. Thus, the design rationality of antenna size is verified.

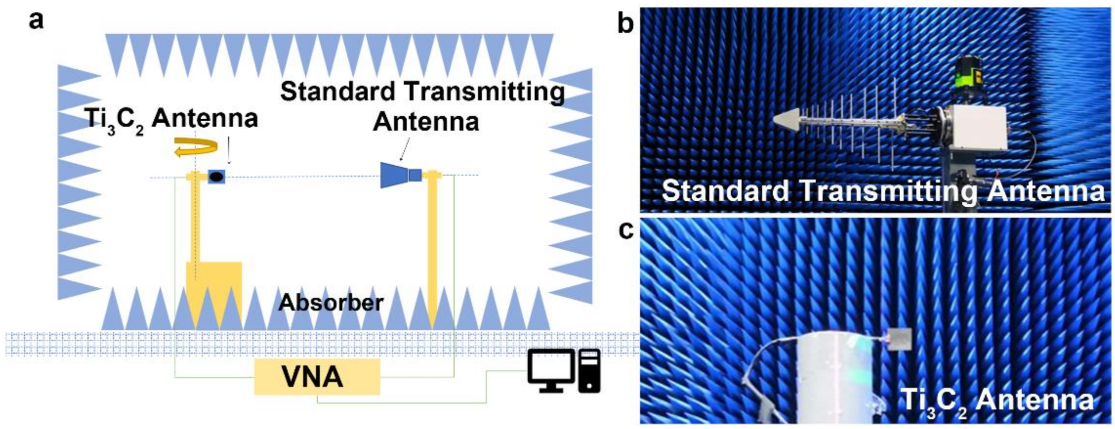

Supplementary Fig. 13 | Schematic illustrations of testing methods for Ti_3C_2 antennas. **a** Gain and radiation pattern measurement in an anechoic chamber. **b** Photograph of standard transmitting antenna. **c** Photograph of Ti_3C_2 antenna.

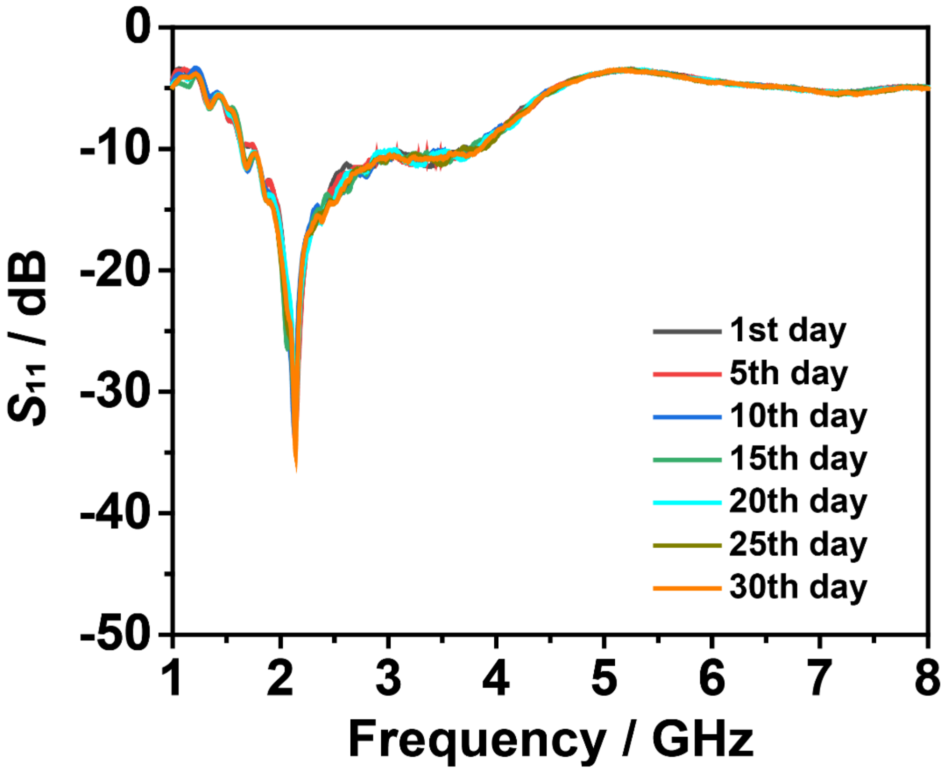

Supplementary Fig. 14 | The reflection coefficient S_{11} repeatable for one month.

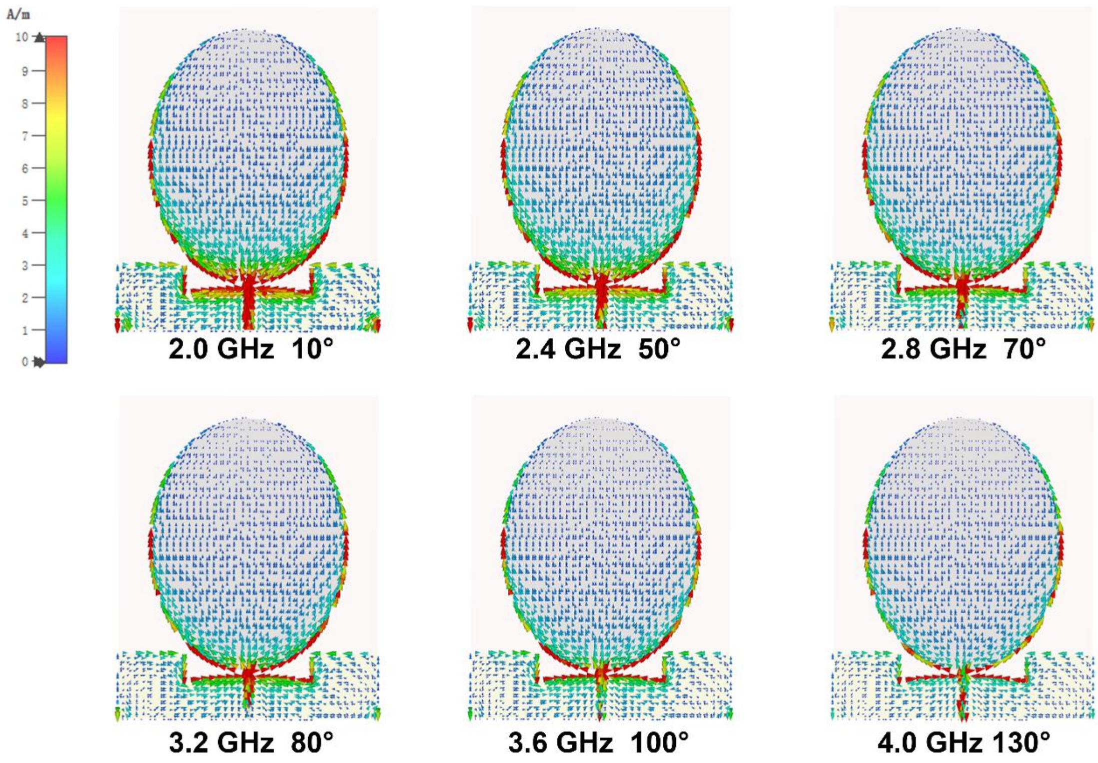

Supplementary Fig. 15 | Current distribution diagram of Ti_3C_2 antenna under different operating frequencies of 2.0, 2.4, 2.8, 3.2, 3.6, and 4.0 GHz, respectively.

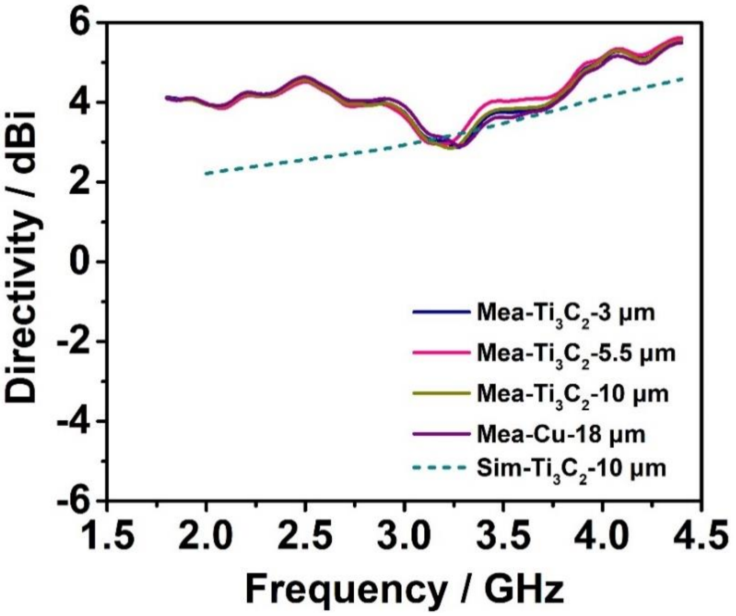

Supplementary Fig. 16 | Measured and simulated directivity of Ti_3C_2 and copper antennas.

The directivity of an antenna is equal to the ratio of the maximum power density to its average value over a sphere as observed in the far field of an antenna.[4] The gain of an antenna is an actual or realized quantity which is less than the directivity due to ohmic losses in the antenna. The relationship between directivity and gain can be derived according to the equation (S1),

$$D = G / k \qquad \text{(S1)}$$

where D is the directivity of the antenna, G is the gain of the antenna, k is the radiation efficiency of the antenna. The difference in directivity between Ti_3C_2 antennas and copper antennas is very small. The results show that using Ti_3C_2 instead of copper does not cause the decreased radiation angle of the antenna. The measured directivity is slightly larger than the simulated directivity, which is due to the influence of metal connector.

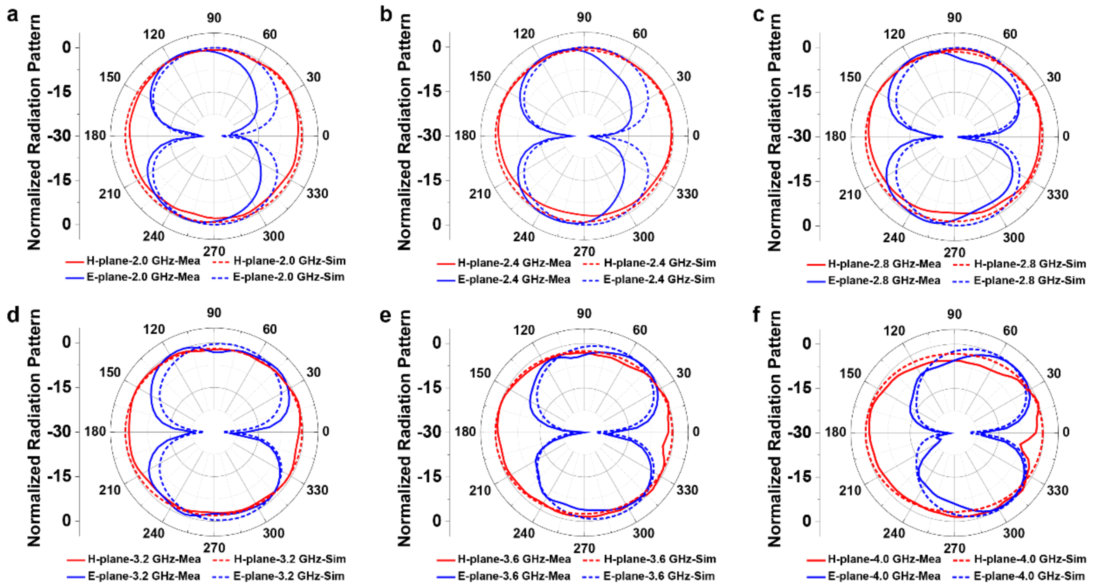

Supplementary Fig. 17 | Comparison of measured and simulated normalized radiation patterns (E/H plane) of antenna under different operating frequencies. **a** 2.0 GHz, **b** 2.4 GHz, **c** 2.8 GHz, **d** 3.2 GHz, **e** 3.6 GHz, **f** 4.0 GHz, respectively.

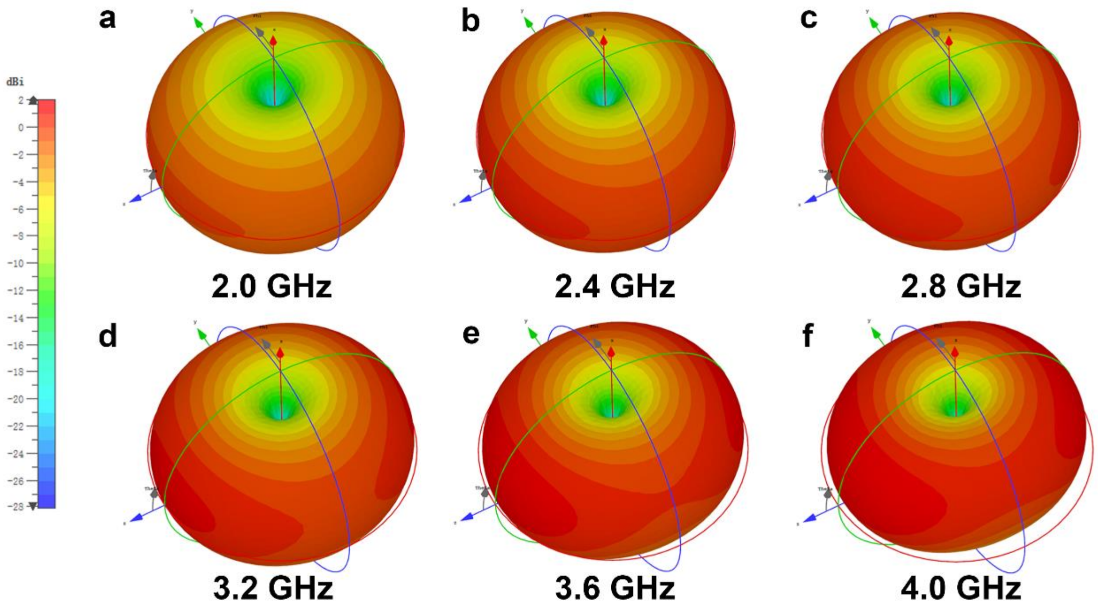

Supplementary Fig. 18 | Simulated 3D radiation patterns of Ti_3C_2-5.5 μm antennas at different frequencies. **a** 2.0 GHz, **b** 2.4 GHz, **c** 2.8 GHz, **d** 3.2 GHz, **e** 3.6 GHz, **f** 4.0 GHz, respectively.

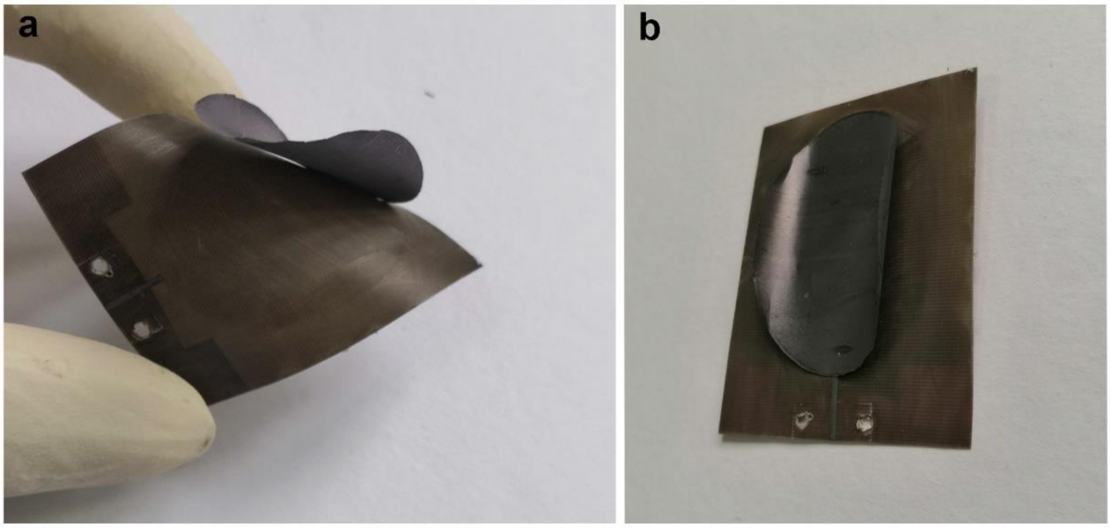

Supplementary Fig. 19 | The optical photograph of Ti_3C_2 antennas without PDA adhesive layer. **a** Ti_3C_2 film is ripped with scotch tape. **b** Ti_3C_2 film sheds after being bent at random angles. It demonstrates the structural instability of Ti_3C_2 antennas without PDA adhesive layer.

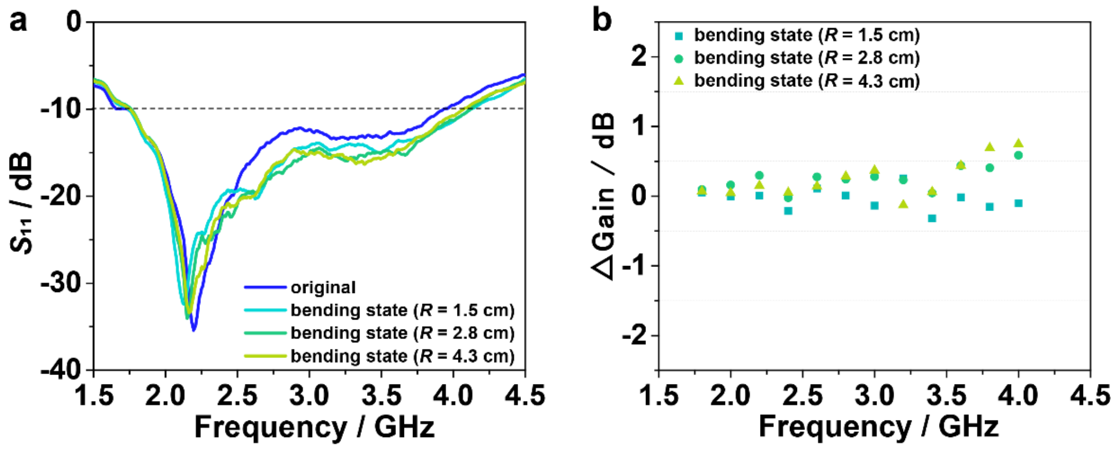

Supplementary Fig. 20 | Flexible Ti_3C_2-5.5 μm antennas. a S_{11} parameter and **b** gain differences of Ti_3C_2 antennas in the bending state under different bending radii.

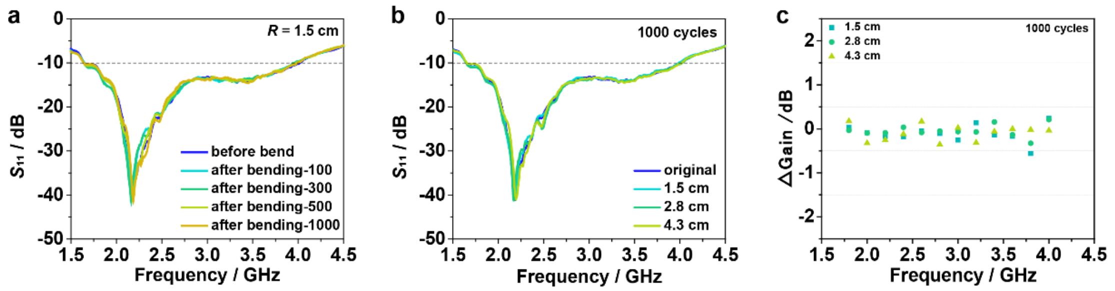

Supplementary Fig. 21 | Flexible Ti_3C_2-3 μm antennas. S_{11} parameter of Ti_3C_2-3 μm antennas after different bending cycles under the radius of 1.5 cm (**a**) and after 1000 bending cycles under different bending radii (**b**). **c** Gain differences of Ti_3C_2-3 μm antennas after 1000 bending cycles under different bending radii.

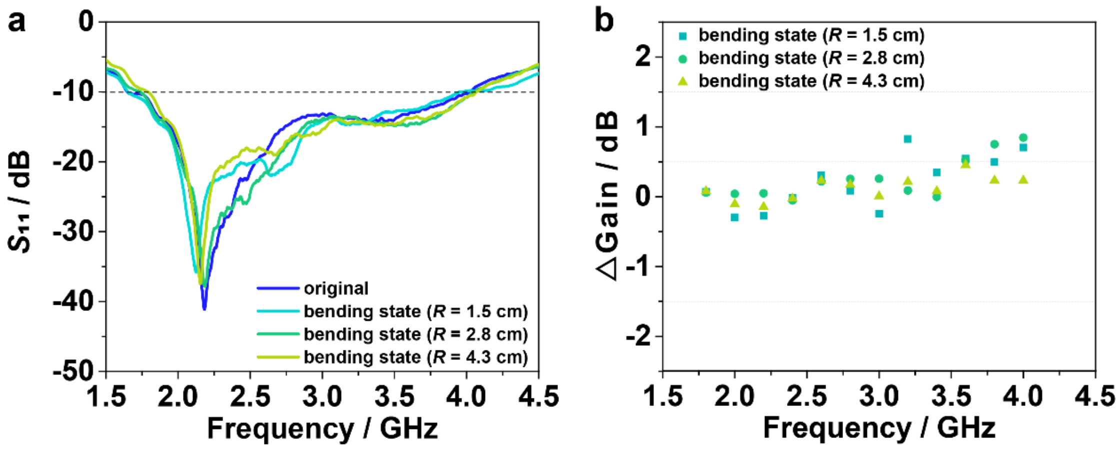

Supplementary Fig. 22 | Flexible Ti_3C_2-3 μm antennas. a S_{11} parameter and **b** gain differences of Ti_3C_2 antennas in the bending state under different bending radii.

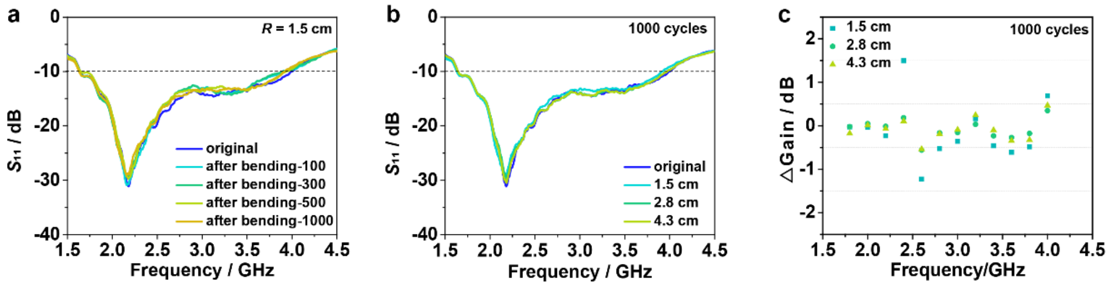

Supplementary Fig. 23 | Flexible Ti_3C_2-10 μm antennas. S_{11} parameter of Ti_3C_2-10 μm antennas after different bending cycles under the radius of 1.5 cm (**a**) and after 1000 bending cycles under different bending radii (**b**). **c** Gain differences of Ti_3C_2-10 μm antennas after 1000 bending cycles under different bending radii.

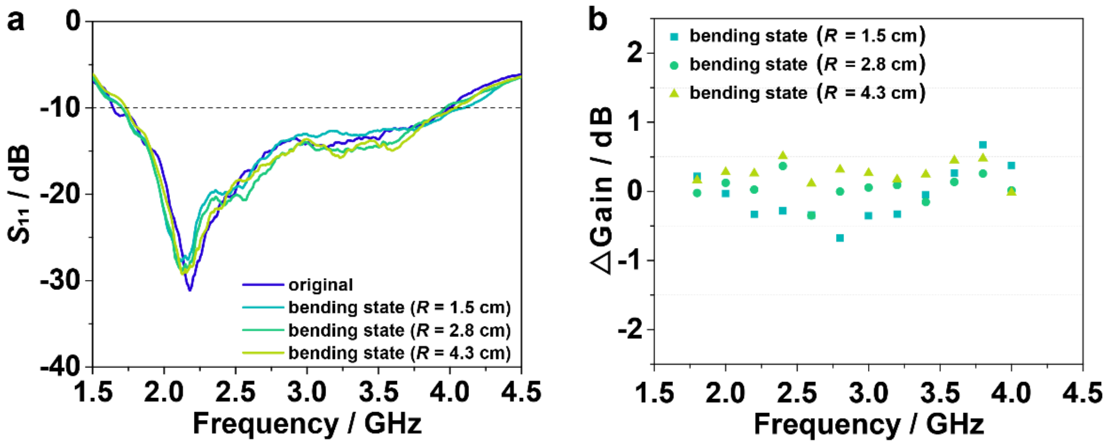

Supplementary Fig. 24 | Flexible Ti_3C_2-10 μm antennas. a S_{11} parameter and **b** gain differences of Ti_3C_2 antennas in the bending state with different bending radii.

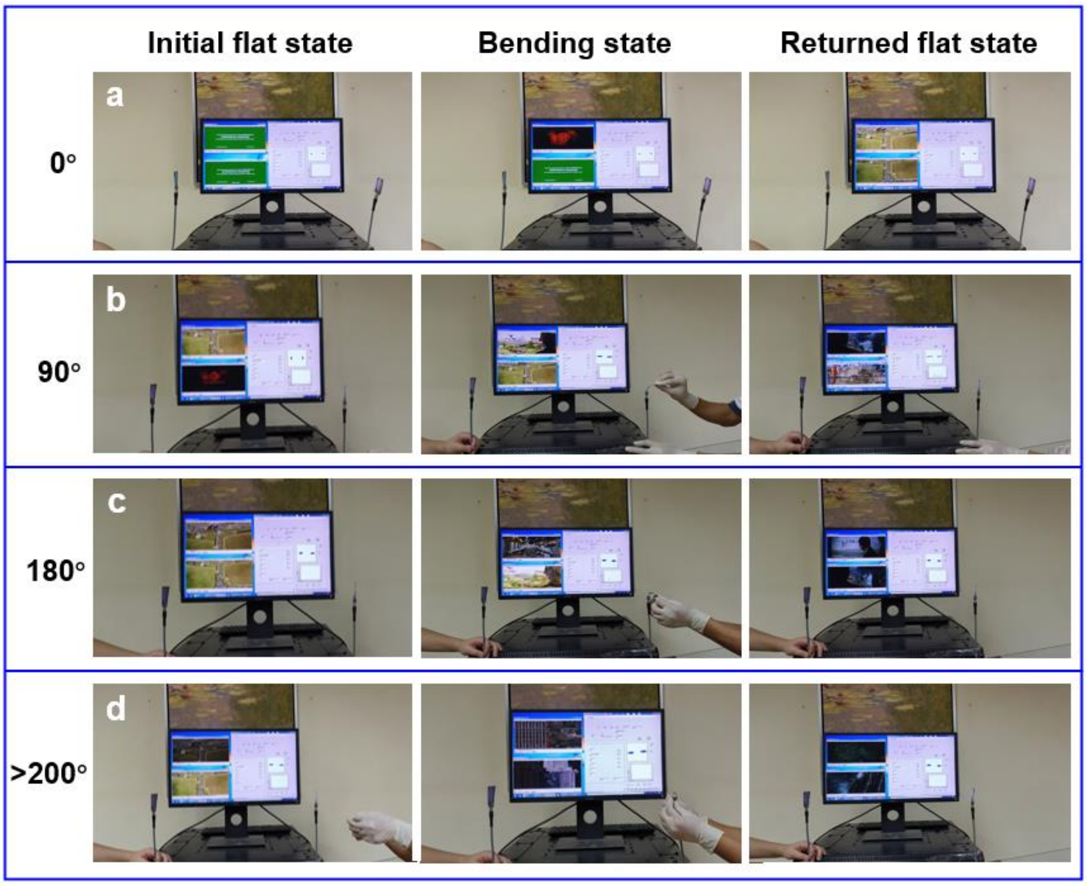

Supplementary Fig. 25 | Real-time movie transmission of Ti_3C_2 antennas under the flat states or different bending angles of (**a**) 0º, (**b**), 90º, (**c**), 180º, and (**d**), > 200º.

The bending radii effects of Ti_3C_2 antennas have been systematically tested under different bending angles for the real-time information transmission and reception (Supplementary Fig. 25 and Supplementary Movie 4). When the transmitting antenna and receiving antenna are both in flat state, the movie transmission is very stable and fluent (Supplementary Fig. 25a). When the receiving antenna is bent at 90°, the movie transmission can be well maintained (Supplementary Fig. 25b). As the receiving antenna is bent at 180°, the movie transmission is slightly weakened (Supplementary Fig. 25c). When the maximum bending angle (> 200º) is reached, the movie signal

becomes seriously delayed, but the signal transmission can still be realized (Supplementary Fig. 25d). The reason for this phenomenon is that the radiation pattern and current distribution of the antennas is distorted as the bending angle increases, which leads to the attenuated movie transmission. Significantly, the transmission performance can return to the initial fluent state after the antenna returns from the bent state to the flat state.

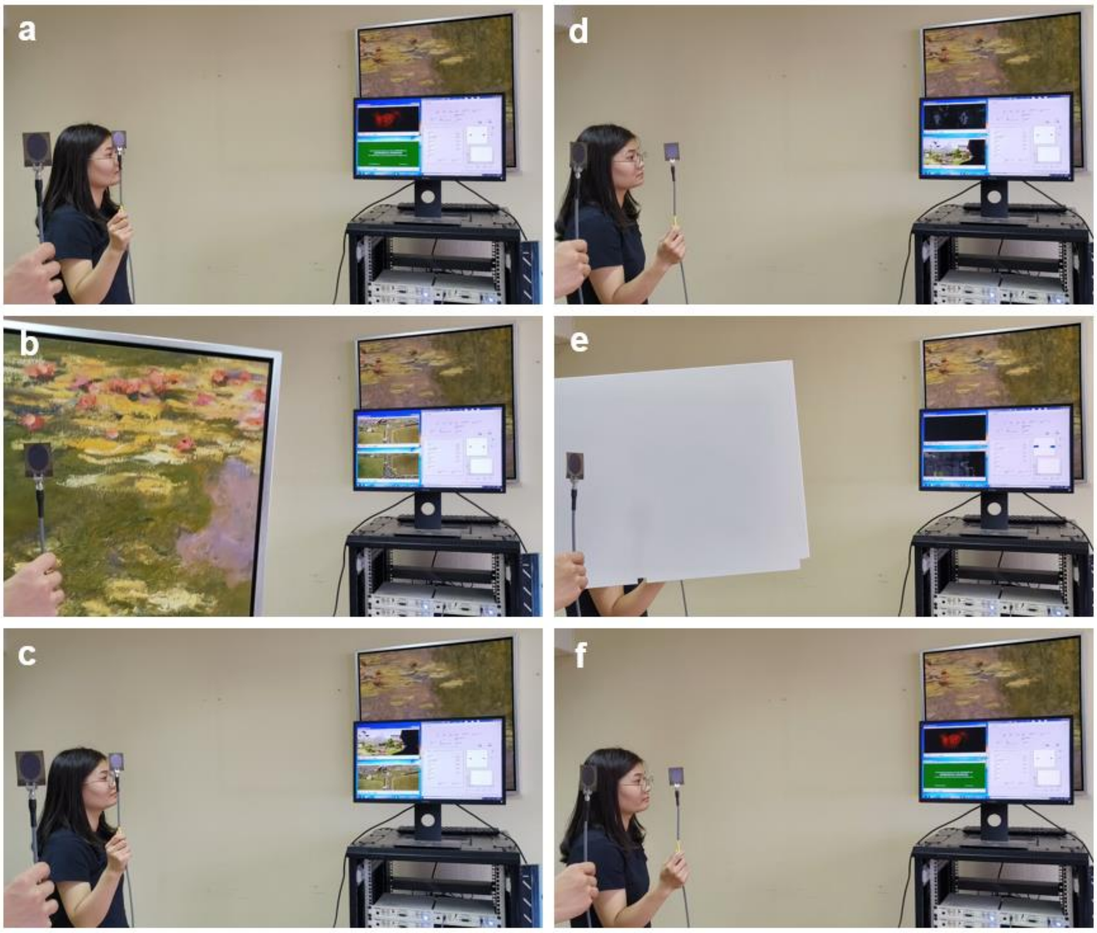

Supplementary Fig. 26 | Real-time movie transmission when two Ti_3C_2 antennas are in the non-line of sight. **a-c** Separated by oil painting, **d-f** Separated by metal plate.

The antenna communication can be achieved when they are in the non-line of sight (Supplementary Fig. 26 and Supplementary Movie 5). The transmitting antenna is set in left side, and the receiving antenna is set in right side (Supplementary Fig. 26a). When they are in the line of sight, the movie transmission is very stable. When the oil painting is used to separate the two antennas, the movie transmission effect is not affected and remains very stable (Supplementary Fig. 26b,c). When the two antennas are separated by a metal plate, the movie transmission suddenly deteriorates for a short time (Supplementary Fig. 26d,e). However, due to the multipath effect in the mobile

communication environment, the movie can be transmitted after a few seconds. After removing the metal plate, the movie transmission becomes stable again (Supplementary Fig. 26f).

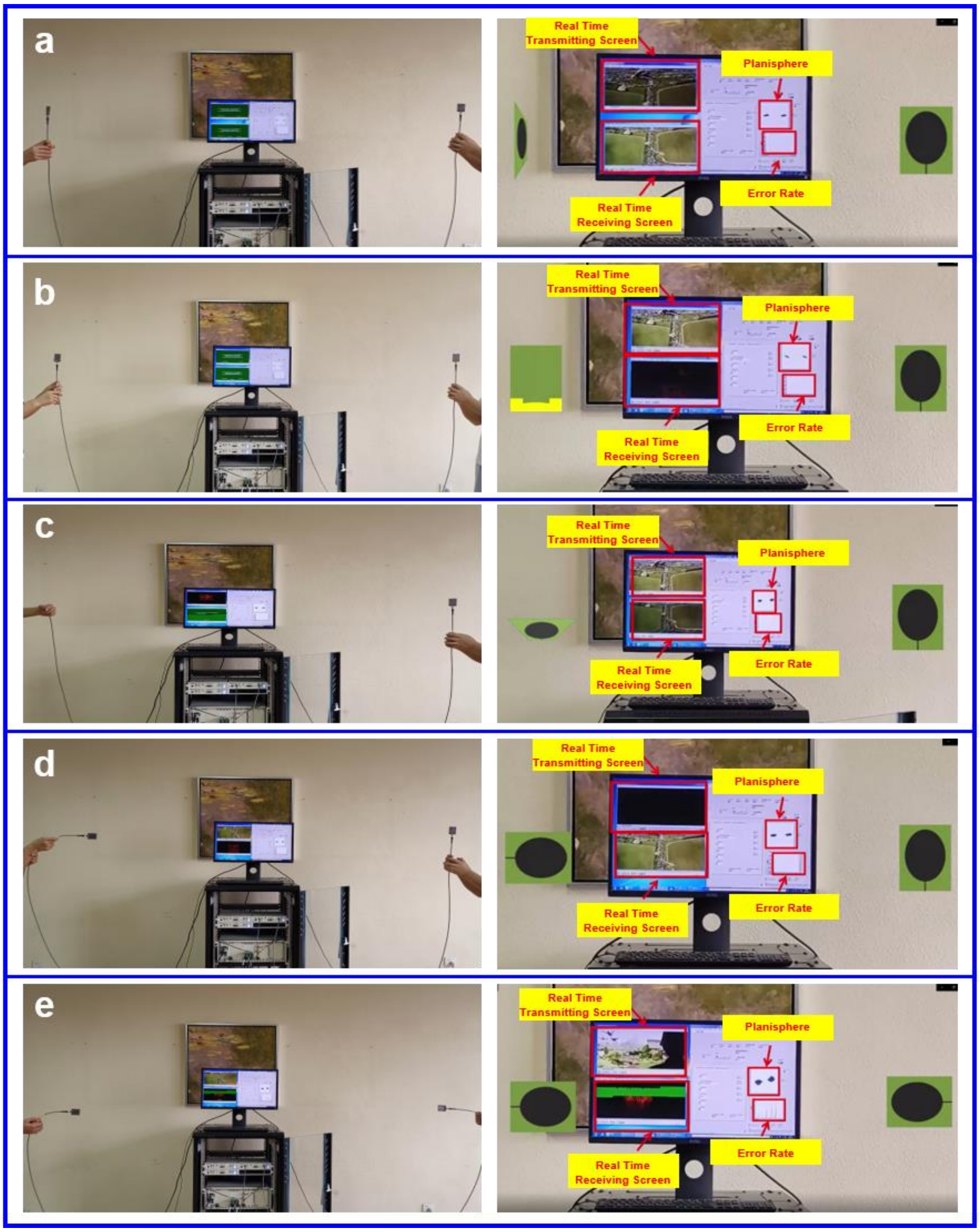

Supplementary Fig. 27 | Real-time movie transmission when two Ti_3C_2 antennas have different orientation angles. a The side of one antenna versus the front of another antenna. **b** The back of one antenna versus the front of another antenna. **c** The transverse direction of one antenna versus the forward direction of another antenna. **d** The head of an antenna versus the side of another antenna. **e** The head of an antenna versus the head of another antenna.

The antennas can also communicate at different orientation angles (Supplementary Fig. 27 and Supplementary Movie 6). When the side of one antenna is versus the front of another antenna (Supplementary Fig. 27a) or the back of one antenna is versus the front of another antenna (Supplementary Fig. 27b), the movie transmission is very stable. When the transverse direction of one antenna is versus the forward direction of another antenna (Supplementary Fig. 27c), the movie transmission becomes slightly worse. This phenomenon is caused by the different polarization directions of the two antennas. When the head of an antenna is versus the side of another antenna (Supplementary Fig. 27d), the movie transmission further deteriorates. This phenomenon is caused by the fact that the polarization direction of the two antennas is different and the maximum gain direction is not aligned. When the head of an antenna is versus the head of another antenna (Supplementary Fig. 27e), the movie transmission almost stops working. The reason for this phenomenon is that both antennas are aligned in the direction of minimum gain.

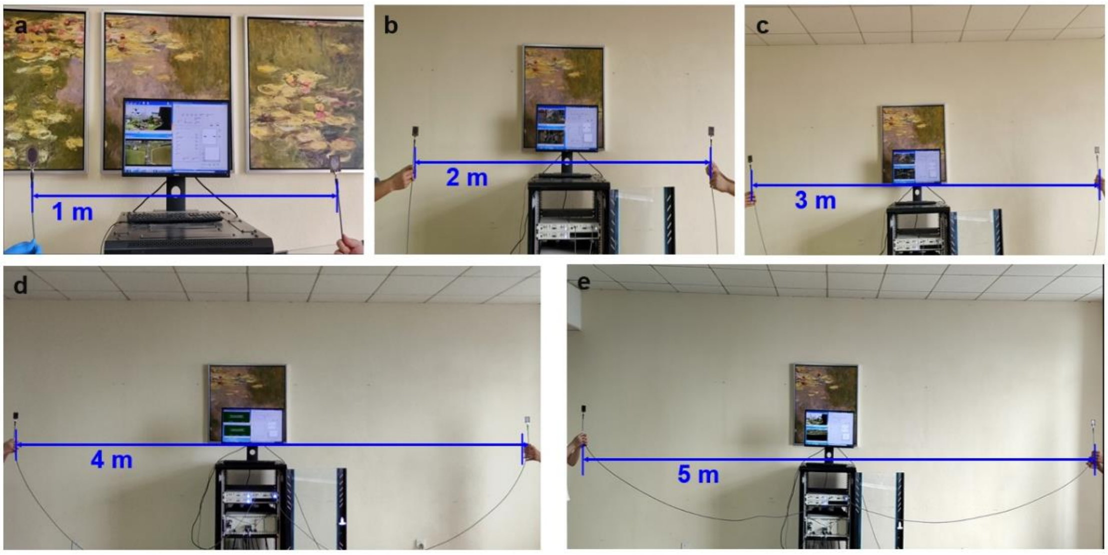

Supplementary Fig. 28 | Real-time movie transmission when two Ti_3C_2 antennas are in the range of meters. a 1 m, **b** 2 m, **c** 3 m, **d** 4 m, **e** 5 m.

The real-time communication can also happen in the long-range distance of 1-5 m (Supplementary Fig. 28 and Supplementary Movie 7). In the experimental setup, the left antenna is the transmitting antenna, and the right antenna is the receiving antenna. When the distance is less than 4 m, the transmission effect of the antenna is very fluent, the points on the planisphere are very concentrated, the error rate is almost 0, and the movie transmission is very stable (Supplementary Fig. 28a-d). When the distance is extended to 5 m, the movie transmission becomes slightly unstable, but the overall transmission effect is still acceptable (Supplementary Fig. 28e).

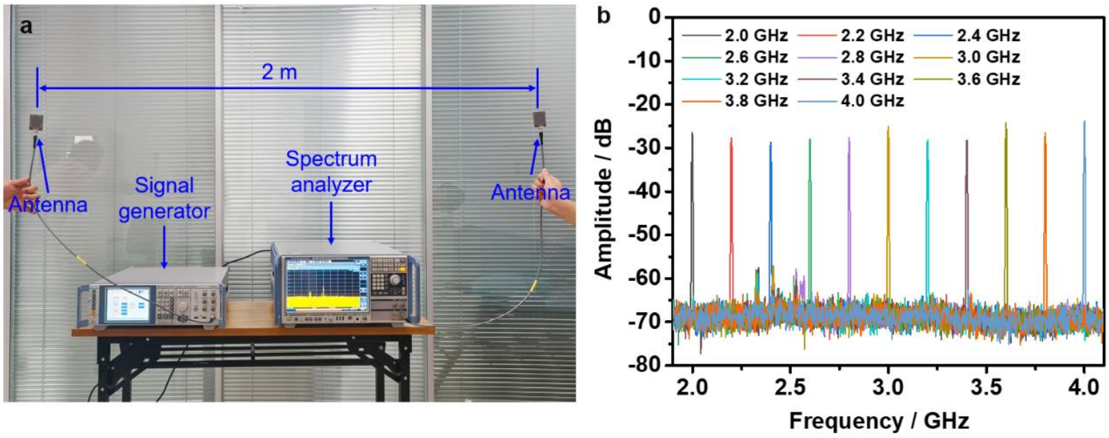

Supplementary Fig. 29 | The communication response in a spectrum analyzer. **a** The experimental setup. **b** Transmission measurement results.

The communication between the antennas where one antenna transmits a signal generated by the signal generator and the receiving antenna reveals the response has been revealed in a spectrum analyzer. The transmitting and receiving antennas are both Ti_3C_2 antennas fabricated by us. The distance between the two antennas is 2 m. The signal generator produces 2-4 GHz signals with 0.2 GHz step and the signal power is 20 dBm. The receiving power of the antenna on the spectrum analyzer can be further calculated according to the free space path loss equation (S2) and Friis equation (S3),[5]

$$L_{path} = 32.45 + 20\lg r + 20\lg f \qquad (S2)$$

where r represents the distance between the two antennas, and f represents the operating frequency of two antennas.

$$P_r = [P_t - L_{path} + 10\lg G_r + 10\lg G_t] \qquad (S3)$$

where P_r represents the receiving power, P_t represents the transmitting power, $10\lg G_r$ represents the gain of the receiving antenna, $10\lg G_t$ represents the gain of the transmitting antenna.

For example, the free space path loss is 48.01 dB at 3.0 GHz, and the gains of the receiving antenna and the transmitting antenna are both 2 dBi. Thus, the receiving power on the spectrum analyzer can be calculated as -24.01 dBm. In addition, the transmission line and the connector will have about 1.5 dB loss in the actual measurement, so the actual receiving power should be -25.51 dBm. In addition, there are some noises of measurement results on Industrial Scientific Medical band caused by wireless systems nearby.

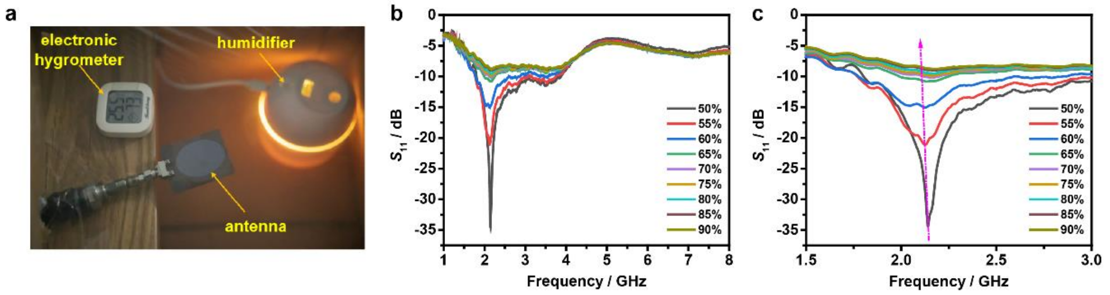

Supplementary Fig. 30 | The humidity effect on the antenna performance. a Humidity sensing experimental setup. **b-c** The antenna sensor response to different concentrations of humidity.

When the humidity is less than 60%, the bandwidth of the antenna remains unchanged, and the amplitude of the resonance point of S_{11} increases sharply. When the humidity is greater than 60%, the bandwidth of the antenna becomes smaller and the S_{11} curve changes slowly. As the humidity further increases to 90%, the amplitude of the S_{11} curve of the antenna correspondingly increases, and the resonance point takes a left-shift. The shift in the resonant frequency of the antenna sensor can be attributed to two reasons. (i) The penetration of water molecules between the Ti_3C_2 nanosheets widens the interlayer spacing, which consequently results in the increased resistivity of the Ti_3C_2 membrane.[6-8] The surface loss of Ti_3C_2 antenna increases, which further results in the decreased Q value.[9] (ii) Due to the humidity change, the dielectric constant of the ambient environment changes, which results in the serious mismatch between the antenna and the free space impedance.

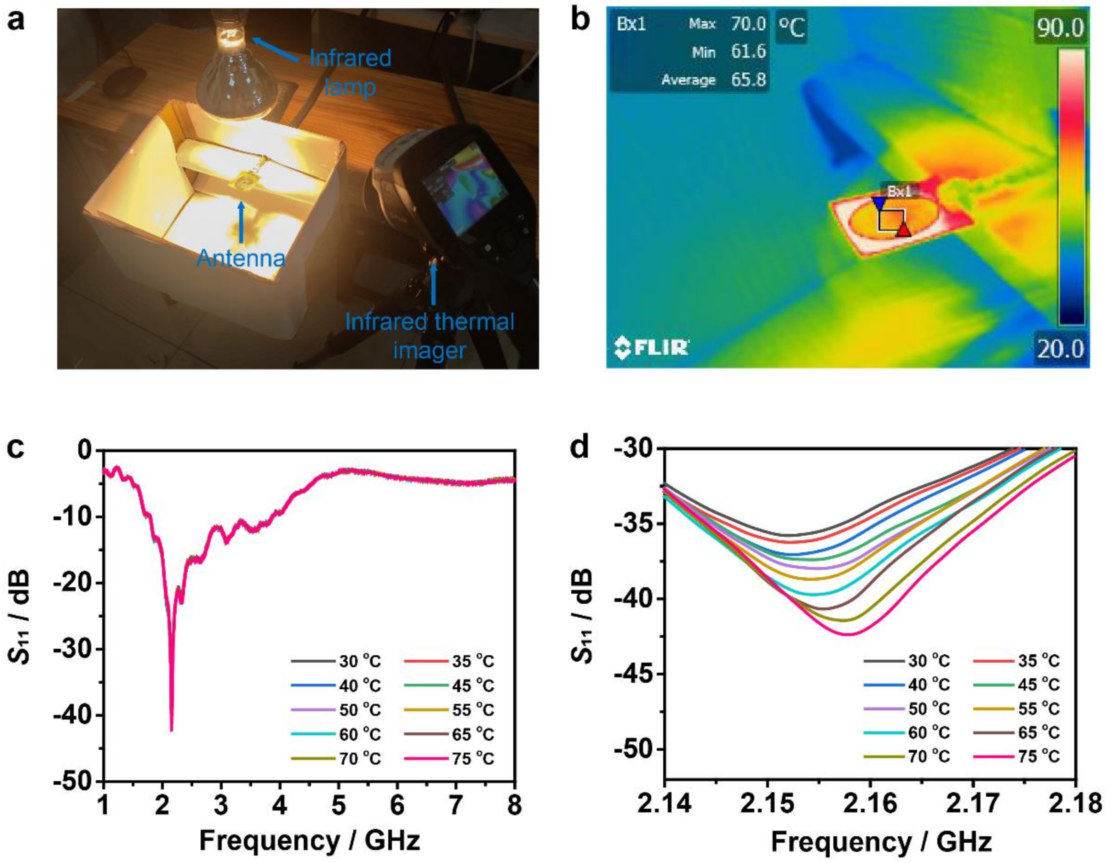

Supplementary Fig. 31 | The heat effect on the antenna performance. a The heat effect experimental setup. **b** Recording picture of thermal imager. **c-d** The antenna sensor response to different temperatures.

As the temperature of the antenna rises, the amplitude of S_{11} curve decreases and the resonance point takes a right-shift (Supplementary Fig. S31c,d). It successfully demonstrates the potentials of the proposed Ti_3C_2 antenna sensor in detecting the temperature of 30-75 °C. The shift in the resonant frequency of the antenna sensor can be attributed to two reasons. (i) Ti_3C_2 possesses excellent photothermal conversion capability. Under near-infrared radiation, the enhanced photothermal effect of Ti_3C_2 results in the increased temperature, which increases the electrical conductivity of Ti_3C_2 film.[10-12] Thus, the surface loss of the antenna decreases, and Q value increases, which

results in the downward shift of the resonant points.[9] (ii) If Ti_3C_2 film is modeled as a parallel lossy resonant circuit, the increased conductivity will lead to the decreased resistive component, which reduces the impedance mismatching losses among the Ti_3C_2 membrane, the antenna feed structure, and the air medium, thus causing the right-shift of the resonant frequency.[7,13]

Supplementary Tables

Supplementary Table 1. The thicknesses of different layers in Ti_3C_2 microstrip TL.

Material	Ti_3C_2	PDA	PET	Adhesive tape	Dielectric substrate	Copper	ENIG	
Thickness [μm]	3.0/5.5/10.0	0.2	—	—	254 (F4B)	18	—	This work
	1.0/3.2/5.5	—	100	100	600 (FR4)	35	5	14

Supplementary Table 2. The thickness, conductivity, and skin depth (at 2.4 GHz) of Ti_3C_2 antenna.

Thickness / μm	Conductivity / S cm^{-1}	Skin depth / μm
3	7765.84	11.66
5.5	7221.39	12.08
10	4474.07	15.36

The skin depth is calculated by the Equation (S4),

$$\delta = \sqrt{1/\pi\sigma\mu f} \qquad \text{(S4)}$$

where σ, μ, and f are conductivity, permeability, and frequency, respectively.[15]

Supplementary Table 3. The quantitative performance and preparation method of patch antennas made of different materials.

Materials	Thickness [μm]	Efficiency [%]	Frequeny [f_0; GHz]	Substrate	Substrate thickness [mm]	Conductivity [S cm^{-1}]	Gain [dBi]	Method	Ref.
Graphene	10	60	4.8	Kapton	0.076	/	2.3	Screen printing	16
Graphene	25	64.9	6	PDMS	2	/	/	Low temperatu re fabricatio n process	17
Cu mesh	20	49-56.88	2.4-2.5	Acrylic plate	1.2	10000	2.65	Vacuum evaporatio n	18
Copper	35	50.93	2.5	Acrylic plate	1.2	10000	4.77	Vacuum evaporatio n	18
Cu mesh	5	42.69	2.45	Acrylic	1	/	2.63	Physical vapor deposition	19
IZTO/Ag/ IZTO	0.1	7.76	2.45	Acrylic	1	/	-4.23	Physical vapor deposition	19
Ag/Ni/Cu fabric	130	58.6	2.45	PDMS	3	/	4.16	Multilayer printing	20
Silver nanoparticle	3	11	2.45	Cardboard	0.56	200000	1	Inkjet-printing	21
Silver paste	26.52	2.81	2.45	NinjaFlex	1.2	170	-7.2	3D printing	22
Silver nanowire	500	41.83	2.92	PDMS	1	8130	4.9	Screen printing	23
Silver	3	31.6	1.89	Kapton	0.1	/	2.2	Inkjet-printing	24
EGaIn	100	45-60	3.43	PDMS	1	34000	/	Injecting method	25
EGaIn	1500	75	5.2	Photopoly-mer resin	6	51000	/	3D printing	26
Ti_3C_2	1.0<br>3.2	80-90.4<br>87-98.4	5.6<br>10.9	RT 5880	1.6	15000	5.74<br>7.14	Spray coating	14

	5.3	90.6-99	16.4				5.48		
Ti_3C_2	20	/	1.841	Rogers 6010	1.91	4200	1.9	Vacuum-assisted filtration	27
Ti_3C_2	3	/	1.834	Rogers 6010	1.91	/	2	Vacuum-assisted filtration	7
	3	68.4				7765.84	3.14		
Ti_3C_2	5.5	68.7	2.45	F4B	0.254	7221.39	3.04	Extrusion printing technology	This work
	10	76.5				4474.07	3.23		

*EGaIn: Eutectic Gallium Indium (liquid metal); PET: Polyethylene Terephthalate; IZTO: In–Zn–Sn–O.

Supplementary Reference

1 O'Bryan, C. S., Brady-Miné, A., Tessmann, C. J., Spotz, A. M. & Angelini, T. E. Capillary forces drive buckling, plastic deformation, and break-up of 3D printed beams. *Soft Matter* **17**, 3886-3894 (2021).

2 Honda, S., Ito, M., Seki, H. & Jinbo, Y. A disk monopole antenna with 1:8 impedance bandwidth and omnidirectional radiation pattern, *Proc. Int. Symp. Antennas Propag.* **4**, 1145 (1992)

3 Agrawall, N. P., Kumar, G. & Ray, K. Wide-band planar monopole antennas. *IEEE Trans. Antennas Propag.* **46**, 294-295 (1998).

4 Kraus, J. D. & Marhefka, R. J. *Antenna: For All Applications (Third Edition)* (Publishing House of Electronics Industry, Beijing, 2011).

5 Friis, H. T. A note on a simple transmission formula. *Proc. IRE* **34**, 254-256 (1946).

6 Shao, Y. et al. Room-temperature high-precision printing of flexible wireless electronics based on MXene inks. *Nat. Commun.* **13**, 3223 (2022).

7 Khorsand Kazemi, K. et al. Low-profile planar antenna sensor based on $Ti_3C_2T_x$ MXene membrane for VOC and humidity monitoring. *Adv. Mater. Interfaces* **9**, 2102411 (2022).

8 An, H. et al. Water sorption in MXene/polyelectrolyte multilayers for ultrafast humidity sensing. *ACS Appl. Nano Mater.* **2**, 948-955 (2019).

9 Belohoubek, E. & Denlinger, E. Loss considerations for microstrip resonators. *IEEE Trans. Microw. Theory Tech.* **23**, 522-526 (1975).

10 Chen, Y. et al. A portable multi-signal readout sensing platform based on plasmonic

MXene induced signal amplification for point of care biomarker detection. *Sens. Actuat. B Chem.* **352**, 131059 (2022).

11 Li, Y. et al. Scalable manufacturing of flexible, durable $Ti_3C_2T_x$ MXene/polyvinylidene fluoride film for multifunctional electromagnetic interference shielding and electro/photo-thermal conversion applications. *Compos. Part B: Eng.* **217**, 108902 (2021).

12 Wang, X. et al. A lightweight MXene-coated nonwoven fabric with excellent flame retardancy, EMI shielding, and electrothermal/photothermal conversion for wearable heater. *Chem. Eng. J.* **430**, 132605 (2022).

13 Wiltshire, B. D. et al. High-frequency TiO_2 nanotube-adapted microwave coplanar waveguide resonator for high-sensitivity ultraviolet detection. *ACS Appl. Mater. Interfaces* **14**, 6203-6211 (2022).

14 Han, M. et al. Solution-processed $Ti_3C_2T_x$ MXene antennas for radio-frequency communication. *Adv. Mater.* **33**, 2003225 (2021).

15 Sarycheva, A. et al. 2D titanium carbide (MXene) for wireless communication. *Sci. Adv.* **4**, eaau0920 (2018).

16 Lamminen, A. et al. Graphene-flakes printed wideband elliptical dipole antenna for low-cost wireless communications applications. *IEEE Antennas Wirel. Propag. Lett.* **16**, 1883-1886 (2017).

17 Elmobarak, H. A. et al. Assessment of multilayered graphene technology for flexible antennas at microwave frequencies. *Microw. Opt. Techn. Lett.* **59**, 2604-2610 (2017).

18 Kang, S. H. & Jung, C. W. Transparent patch antenna using metal mesh. *IEEE Trans.*

Antennas Propag. **66**, 2095-2100 (2018).

19 Hong, S., Kim, Y. & Jung, C. W. Transparent microstrip patch antennas with multilayer and metal-mesh films. *IEEE Antennas Wirel. Propag. Lett.* **16**, 772-775 (2017).

20 Simorangkir, R. B. V. B., Yang, Y., Matekovits, L. & Esselle, K. P. Dual-band dual-mode textile antenna on pdms substrate for body-centric communications. *IEEE Antennas Wirel. Propag. Lett.* **16**, 677-680 (2017).

21 Saghlatoon, H., Sydänheimo, L., Ukkonen, L. & Tentzeris, M. Optimization of inkjet printing of patch antennas on low-cost fibrous substrates. *IEEE Antennas Wirel. Propag. Lett.* **13**, 915-918 (2014).

22 Rizwan, M., Khan, M. W. A., Sydänheimo, L., Virkki, J. & Ukkonen, L. Flexible and stretchable brush-painted wearable antenna on a three-dimensional (3D) printed substrate. *IEEE Antennas Wirel. Propag. Lett.* **16**, 3108-3112 (2017).

23 Song, L., Myers, A. C., Adams, J. J. & Zhu, Y. Stretchable and reversibly deformable radio frequency antennas based on silver nanowires. *ACS Appl. Mater. Interfaces* **6**, 4248-4253 (2014).

24 Alford Chauraya et al. Inkjet printed dipole antennas on textiles for wearable communications. *IET Microw. Antennas Propag.* **7**, 760-767 (2013).

25 Hayes, G. J., So, J., Qusba, A., Dickey, M. D. & Lazzi, G. Flexible liquid metal alloy (EGaIn) microstrip patch antenna. *IEEE Trans. Antennas Propag.* **60**, 2151-2156 (2012).

26 Huang, G., Liang, J., Zhao, L., He, D. & Sim, C. Package-in-dielectric liquid patch

antenna based on liquid metal alloy. *IEEE Antennas Wirel. Propag. Lett.* **18**, 2360-2364 (2019).

27 Khorsand Kazemi, K. et al. MXene membrane in planar microwave resonant structures for 5G applications. *Appl. Mater. Today* **26**, 101294 (2022).
