# Supplementary material for: 2D Titanium carbide printed flexible ultrawideband monopole antenna for wireless communications
Source: Nat Commun. 2023 Jan 17;14:278. doi: 10.1038/s41467-022-35371-6 (PMC9845342; doi:10.1038/s41467-022-35371-6)
Supplement: Supplementary file 3 — Description of Additional Supplementary Files [file 41467_2022_35371_MOESM3_ESM.docx]

**Description of Additional Supplementary Files**

**File Name: Supplementary Movie 1**

**Description:** Demonstration of the flexibility of Ti_3_C_2_ antennas.

**File Name: Supplementary Movie 2**

**Description:** The transmission performance of flat Ti_3_C_2_ antenna for transmitting movie trailers.

**File Name: Supplementary Movie 3**

**Description:** The transmission performance of bent Ti_3_C_2_ antenna for transmitting movie trailers.

**File Name: Supplementary Movie 4**

**Description:** Real-time movie transmission of Ti_3_C_2_ antenna under different bending angles.

**File Name: Supplementary Movie 5**

**Description:** Real-time movie transmission when two Ti_3_C_2_ antennas are in the non-line of sight.

**File Name: Supplementary Movie 6**

**Description:** Real-time movie transmission when two Ti_3_C_2_ antennas have different orientation angles.

**File Name: Supplementary Movie 7**

**Description:** Real-time movie transmission when two Ti_3_C_2_ antennas are in the range of meters.
